# Unveiling the paths of COVID-19 in a large city based on public transportation data

**DOI:** 10.1038/s41598-023-32786-z

**Published:** 2023-04-08

**Authors:** Jorge L. B. Araújo, Erneson A. Oliveira, Antonio S. Lima Neto, José S. Andrade, Vasco Furtado

**Affiliations:** 1 Laboratório de Ciência de Dados e Inteligência Artificial Universidade de Fortaleza, Fortaleza, Ceará 60811-905 Brazil; 2 Programa de Pós Graduação em Informática Aplicada Universidade de Fortaleza, Fortaleza, Ceará 60811-905 Brazil; 3 Mestrado Profissional em Ciências da Cidade Universidade de Fortaleza, Fortaleza, Ceará 60811-905 Brazil; 4 Célula de Vigilância Epidemiológica Secretaria Municipal da Saúde, Fortaleza, Ceará 60810-670 Brazil; 5 Centro de Ciências da Saúde Universidade de Fortaleza, Fortaleza, Ceará 60811-905 Brazil; 6 Departamento de Física, Universidade Federal do Ceará, Fortaleza, Ceará 60455-760 Brazil; 7 Empresa de Tecnologia da Informação do Ceará Governo do Estado do Ceará, Fortaleza, Ceará 60130-240 Brazil

**Keywords:** Viral infection, Complex networks

## Abstract

Human mobility plays a key role in the dissemination of infectious diseases around the world. However, the complexity introduced by commuting patterns in the daily life of cities makes such a role unclear, especially at the intracity scale. Here, we propose a multiplex network fed with 9 months of mobility data with more than 107 million public bus validations in order to understand the relation between urban mobility and the spreading of COVID-19 within a large city, namely, Fortaleza in the northeast of Brazil. Our results suggest that the shortest bus rides in Fortaleza, measured in the number of daily rides among all neighborhoods, decreased $\approx 25$% more than the longest ones after an epidemic wave. Such a result is the opposite of what has been observed at the intercity scale. We also find that mobility changes among the neighborhoods are synchronous and geographically homogeneous. Furthermore, we find that the most central neighborhoods in mobility are the first targets for infectious disease outbreaks, which is quantified here in terms of the positive linear relation between the disease arrival time and the average of the closeness centrality ranking. These central neighborhoods are also the top neighborhoods in the number of reported cases at the end of an epidemic wave as indicated by the exponential decay behavior of the disease arrival time in relation to the number of accumulated reported cases with decay constant $\lambda \approx 33$ days. We believe that these results can help in the development of new strategies to impose restriction measures in the cities guiding decision-makers with smart actions in public health policies, as well as supporting future research on urban mobility and epidemiology.

## Introduction

COVID-19 has caused massive loss of lives, economic destabilization, and changes in social relations worldwide^1–3^. In order to mitigate the spread of SARS-CoV-2, several countries have implemented restriction measures, such as curfews and lockdowns, which induce a rupture in human mobility^4–6^. Such rupture yields an immediate control of the number of reported cases, at least for some time, to the detriment of the local economy and the mental health of the population^7–9^. In most cases, restriction measures are asynchronous across national territories due to the phase difference among epidemic waves in each city^10^. Nevertheless, they provide interesting case studies to researchers that aim to understand the mechanisms behind the dynamics of COVID-19 dissemination in order to improve mathematical model predictions for future outbreaks^11,12^.

There is a consensus that human mobility plays a fundamental role in the dissemination of infectious diseases at the intercity scale^13–15^. Brockmann and Helbing^16^ introduced a network model to study the dissemination of 2009 H1N1 flu and 2003 SARS among different cities worldwide. They found that the propagation of infectious diseases among cities is better described by an effective distance (defined in relation to human mobility) than by the geographic distance since the disease arrival time in the cities only correlates with the effective distance to the Initial Outbreak Location (IOL). Therefore, the effective distance is interpreted as the most probable path through which an infectious disease spreads, *i.e.*, the path along which the probability of population mixing is maximized. On the other hand, this role remains unclear at the intracity scale despite the use of sophisticated techniques, such as contact tracing and meta-population models^17–21^, in order to find a reliable correlation between urban mobility and the spreading of infectious diseases. One of the main reasons for this lack of clarity is the rise of complexity introduced by commuting patterns in the daily life of cities.

Here, we perform a longitudinal analysis based on concepts of complex networks fed with 9 months of epidemiological and mobility data in order to understand the relation between urban mobility and the spreading of COVID-19 within Fortaleza, a large Brazilian city. Brazil is the biggest country in Latin America, having a population of 211 million people (estimated for 2020)^22^. Brazil’s first case of COVID-19 was reported on February 26th, 2020^23^. At the end of that year, the accumulated cases and deaths of COVID-19 were 7,680,082 and 195,008^24^, respectively. Regarding Fortaleza, capital of the state of Ceará, a city with a population of 2.68 million people (estimated for 2020), the first case of COVID-19 was officially reported in Meireles neighborhood on March 16th, 2020^25^. Over that year, 87,636 cases and 4980 deaths of COVID-19 in the city were reported^26^. Taking that into account, we introduce a model that uses the flow of individuals among neighborhoods in order to estimate the most probable path in which an infectious disease spreads and, consequently, define the most important neighborhoods from the point of view of commuting patterns. Further, we show how a lockdown influences urban mobility and, consequently, the arrival time of an outbreak at the intracity scale. The manuscript is concluded with a discussion regarding the maintenance of the order in the ranking of the most important neighborhoods throughout the investigated epidemic period. Our main contribution is to find evidence that these most important neighborhoods are not only the primary targets for infectious disease outbreaks, but also the top neighborhoods in the number of reported cases at the end of an epidemic wave. This allows us to shed light on a potentially more effective definition of local restriction measures.

## Results and discussion

### Socio-economical and epidemiological indicators

**Figure 1:**
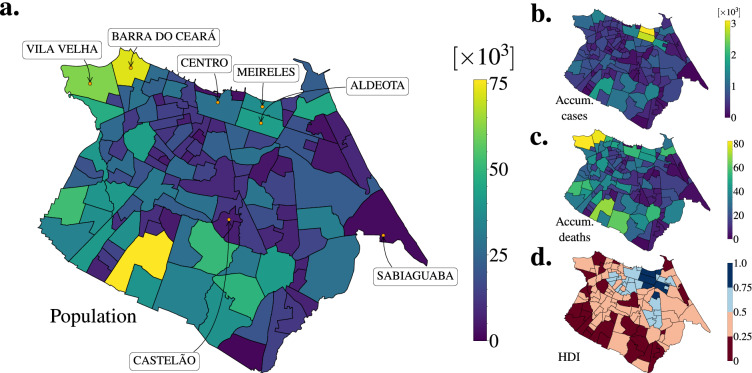
Geographic and social-economical structure of Fortaleza, as well as, some COVID-19 indicators for 2020. (**a**) Spatial distribution of population. (**b**) Spatial distribution of accumulated reported cases of COVID-19. (**c**) Spatial distribution of accumulated deaths of COVID-19. (**d**) Spatial distribution of Human Development Index (HDI). We can realize that there was a sub-notification of reported cases of COVID-19 in Fortaleza. The neighborhoods with the highest HDIs hold the largest numbers of reported cases since their inhabitants had more access to COVID-19 tests in private practices. However, we emphasize that such neighborhoods are not the top ones in deaths. As explained in the main text, we believe that the existing bias is not strong enough to change the conclusions our study. All regional maps were produced using Python packages. The map is from the package Matplotlib Version 3.7 (https://matplotlib.org/).

We show the geographic and social-economical structure of Fortaleza, as well as, some COVID-19 indicators for 2020 in Fig. 1 (see Datasets). Figure 1a shows the spatial distribution of population with the names of neighborhoods that will be mentioned throughout our study. Figure 1b,c show the spatial distribution of accumulated reported cases and deaths of COVID-19, respectively. We also show the spatial distribution of the Human Development Index (HDI) in Fig. 1d. There was a sub-notification of reported cases of COVID-19 in Fortaleza. We can realize such fact by making a comparison among the Fig. 1b,c,d. The neighborhoods with the highest HDIs hold the largest numbers of reported cases since their inhabitants had more access to COVID-19 tests in private practices. However, we emphasize that such neighborhoods are not the top ones in deaths. That being said, we believe that this sub-notification was geographically homogeneous in the city, except for the highest HDI neighborhoods (5 out of 119), since we are able to recover the expected positive linear relation between cases and deaths (see Fig. SI–1). This result suggests the existing bias is not strong enough to change our study.

**Figure 2:**
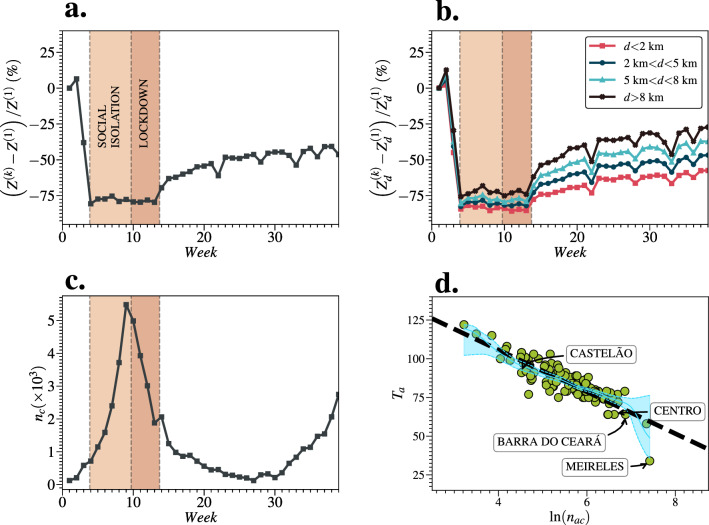
Mobility changes, number of reported cases, and disease arrival time. In (**a**), we show the time evolution of the mobility changes in percentage through the number of rides among all neighborhoods in a given week *k*, $Z^{(k)}=\sum _{ij(i\ne j)} F_{ij}^{(k)}$. It is possible to note a substantial decrease in urban mobility from week 3 (mid-March) to week 14 (end of May) since restriction measures (in brown) were implemented during this period. After these two closing periods, the economic reopening process started, and the mobility slowly returned to the baseline ($Z^{(1)}$). (**b**) shows the same as in (**a**), but stratified by geographic distance *d* through the quantity $Z_d^{(k)}=\sum _{ij(d_{ij} \in d)} F_{ij}^{(k)}$, which is measured by taking into account only the neighborhoods in which the geographic distances among their centroids lie in the range of the stratum *d*. Therefore, we can see that the shortest rides changed $\approx 25$% more than the longest in Fortaleza. This effect is the opposite of what is observed on a higher geographic scale (intercity regime)^10^. As shown in (**c**), the restriction measures were essential to reduce the number of cases ($n_{c}$) in Fortaleza. In (**d**), we find that the number of accumulated reported cases (until the end of the studied period) $n_{ac}$ decays exponentially with the arrival time $T_a$ of the disease in the neighborhoods, $n_{ac} \sim \exp (-T_a/\lambda )$, with the decay constant $\lambda \approx 33$ days ($r^{2}\approx 0.73$). Here, $T_a$ is measured as the number of days from January 1st, 2020 until the day that a threshold of $n_{ac}^{*}=6$ reported cases is reached. The dashed line corresponds to the linear fit between $\ln (n_{ac})$ and $T_{a}$. The solid blue line is the Nadaraya-Watson (NW) estimator, and the two dashed blue lines are its 95% Confidence Intervals (CI) estimated using the bootstrap method.

Figure 2 shows an overview of the COVID-19 spreading across Fortaleza during the studied period. We calculate the mobility changes through the quantity $(Z^{(k)}-Z^{(1)})/Z^{(1)}$, where $Z^{(k)}=\sum _{ij(i\ne j)} F_{ij}^{(k)}$ is the number of rides among all neighborhoods in a given week *k* (see Fig. 2a). Similar to other cities, the restriction measures implemented in Fortaleza were divided into two closing periods and one opening period: (i) a social isolation period (from week 4–9, State Decree 33,519), in which some services (*e.g.*, schools and universities) were closed, and the people were not obligated to stay at home; (ii) a lockdown period (from week 10–13, State Decree 33,574), in which the people were obligated to stay at home, and only essential services were functioning; and (iii) an economic reopening period (from week 14 onwards, State Decree 33,608), in which all services started to reopen, and the people were allowed to leave their houses. In this context, we observe that urban mobility abruptly reduced and gradually increased in Fortaleza when the first restriction measures came into force and when the economic reopening progressed, respectively. Figure 2b shows these changes stratified by geographic distance *d* through a similar ratio $(Z_d^{(k)}-Z_d^{(1)})/Z_d^{(1)}$, where $Z_d^{(k)}=\sum _{ij(d_{ij} \in d)} F_{ij}^{(k)}$. The quantity $Z_d^{(k)}$ is measured by taking into account only the neighborhoods in which the geographic distances among their centroids lie in the range of the stratum *d*. We find that the shortest rides decreased $\approx 25$% more than the longest ones during the studied period, which is the opposite of what has been observed at the intercity scale^10^. Furthermore, we also find evidence that mobility changes and HDI were correlated in Fortaleza (see Fig. SI–2), similar to previous studies^27,28^. The weekly number of reported cases, $n_c$, is shown in Fig. 2c. We observe that the peak of reported cases took place right before the lockdown period. This little anticipation is due to the fact that the dates of reference adopted for the *x*-axis correspond to the days of the onset of symptoms. As shown in Fig. 2d, the number of accumulated reported cases (until the end of the studied period) $n_{ac}$ decays exponentially with the arrival time $T_a$ of the disease in the neighborhoods, $n_{ac} \sim \exp (-T_{a}/\lambda )$, with the decay constant $\lambda \approx 33$ days. Precisely, $T_a$ is measured as the number of days from January 1st, 2020 until the day that a threshold of $n_{ac}^{*}=6$ reported cases is reached (see Supplementary Information). We also perform the calculation of the Nadaraya-Watson estimator to validate our exponential decay hypothesis^29,30^.

### Network metrics and centrality measures

**Figure 3:**
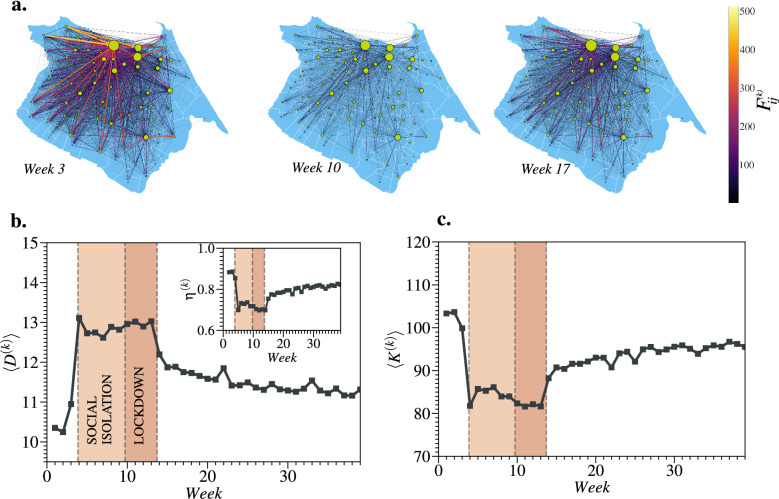
Overview of the multiplex network. In (**a**), we show three layers of the proposed network, corresponding to week 3 (before the restriction measures), week 10 (during the lockdown), and week 17 (after the beginning of the economic reopening). The vertices stand for the neighborhoods of Fortaleza and the weighted directed edges stand for $F_{ij}^{(k)}$, the average daily number of individuals that ride from a neighborhood *i* to another *j* at week *k*. The sizes of the vertices corresponds to their weighted in-degrees (normalized by the average in each layer *k*). The widths/colors of the edges indicate the magnitude of $F_{ij}^{(k)}$ as shown by the color bar in linear scale. The maps show synchronization of mobility changes among the neighborhoods, which is uncommon to find among regions or cities due to different stages of local outbreaks^6,10^. The largest three vertices in the maps are *Centro* (downtown), *Aldeota*, and *Meireles*. Meanwhile, the three highest values of $F_{ij}^{(k)}$ are (*Barra do Ceará*, *Centro*), (*Vila Velha*, *Centro*), and (*Barra do Ceará*, *Meireles*). As the number of rides decreased (see Fig. 2a) during social isolation and lockdown periods, the average shortest path length $\langle D^{(k)} \rangle$ increases by $\approx 30$% as shown in (**b**). The decrease in the number of rides eliminates some connections among neighborhoods, which reduces the density of the network $\eta ^{(k)}$. The average effect of mobility restrictions in the network connections is shown through the average of the in-degrees $\langle K^{(k)} \rangle$, as illustrated in (**c**). After the restriction measures, these metrics tend to return to their original state in the pre-pandemic period. All regional maps were produced using Python packages. The map is from the package Matplotlib Version 3.7 (https://matplotlib.org/ ). Networks are from the package Networkx Version 2.5.1 (https://networkx.org/).

Three layers of the proposed network (see Methods) are shown in Fig. 3a, corresponding to week 3 (before the restriction measures), week 10 (during the lockdown), and week 17 (after the beginning of the economic reopening) during the investigated epidemic wave of COVID-19. The maps show synchronization of mobility changes among the neighborhoods, which is uncommon to find on a larger geographic scale, *e.g.*, among regions or cities, due to different stages of local outbreaks^6,10^. The size of the vertices is based on their weighted in-degrees. The neighborhoods *Centro* (downtown), *Aldeota*, and *Meireles* are the largest three vertices in the maps. Further, the magnitude of the directed edges, which is defined by $F_{ij}^{(k)}$, is compatible with the macroscopic evolution of the mobility shown in Fig. 2a. The edges corresponding to the three highest values of $F_{ij}^{(k)}$ are (*Barra do Ceará*, *Centro*), (*Vila Velha*, *Centro*), and (*Barra do Ceará*, *Meireles*). Figure 3b shows the average shortest path length $\langle D^{(k)}\rangle = [n(n-1)]^{-1}\sum _{ij(i\ne j)} D_{ij}^{(k)}$ for each week *k* of the studied period. As compared with the values observed before the restriction measures, we observe an increase of $\approx 30$% in $\langle D^{(k)}\rangle$ during the social isolation and lockdown periods, due to the substantial decrease in the number of rides in Fortaleza. Such a result indicates that, on average, a hypothetical outbreak would take longer to reach all neighborhoods if the regular urban mobility was similar to the one found during the restriction measures. In the same time interval, the density $\eta ^{(k)} = m/[n(n-1)]$, where *m* is the number of edges in the layer *k*, decreased as shown in the inset of Fig. 3b. Since *n* is constant, such behavior implies missing edges during this period, which is consistent with the rise of the $\langle D^{(k)}\rangle$. In Fig. 3c, the average of the in-degrees $\langle K^{(k)}\rangle$ exhibits a behavior similar to $\eta ^{(k)}$, indicating a decrease in the probability of mixing the populations among neighborhoods during the restriction measures. All metrics tend to eventually return to their original values, *i.e.*, before the restriction period.

### Shortest path distributions

**Figure 4:**
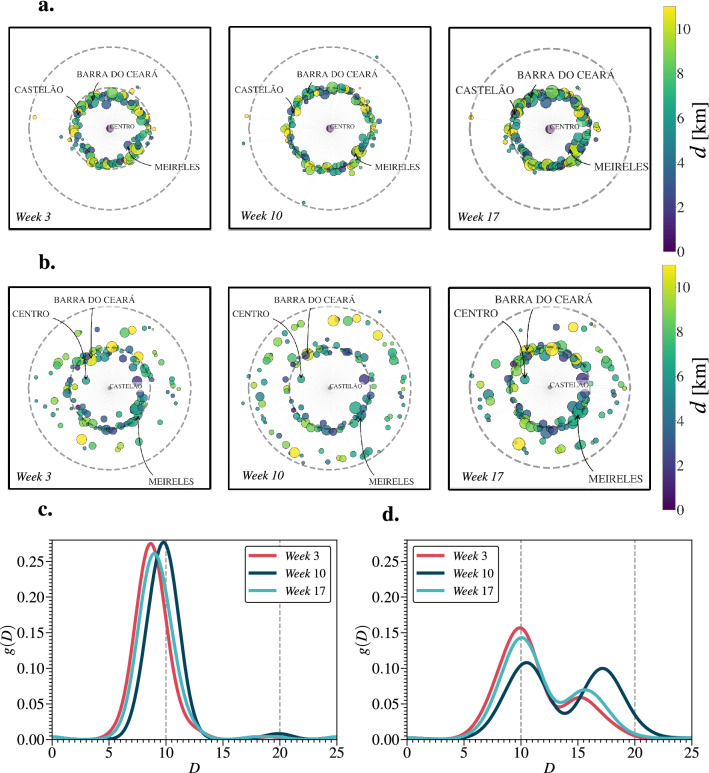
Polar representations and shortest path distributions of $D_{ij}^{(k)}$ for different Initial Outbreak Locations (IOLs). In (**a**) and (**b**), we show the polar representations during weeks 3 (before the restriction measures), 10 (during the lockdown), and 17 (after the beginning of the economic reopening) from two particular IOLs, *Centro* and *Castelão*, to all other neighborhoods of Fortaleza. The value of $D_{ij}^{(k)}$ from *i* to *j* at the week *k* is calculated through the Dijkstra’s algorithm^38^ upon each layer of the multiplex network. The polar representation is a coordinate system, where the IOL is set in as the origin, the radius stands for $D_{ij}^{(k)}$ and the angle stands for a random number between 0 and $2\pi$. Each point represents a neighborhood in which its size is proportional to the population $N_{i}$ and its color is based on the geographic distance $d_{ij}$, measured in kilometers (*km*), according to the color bars in linear scale. The dashed gray lines stand for $D_{ij}^{(k)} = 10$ and $D_{ij}^{(k)} = 20$. In (**c**) and (**d**), we show the radial distributions of the shortest paths $g(D_{ij}^{(k)})$ for the same previous IOLs and weeks. We calculate $g(D_{ij}^{(k)})$ through the Kernel Density Estimation (KDE) with a Gaussian kernel (see Methods). In both cases, $g(D_{ij}^{(k)})$ shifts to the right from week 3–10, showing that there is, on average, an increase in $D_{ij}^{(k)}$, and shifts back to the left from week 10–17, partially recovering the urban mobility pattern observed in week 3. For all other neighborhoods as IOLs, we find similar behaviors, even in the cases where $g(D_{ij}^{(k)})$ has a bimodal-like shape, as shown in (**d**).

The effective distance distributions during weeks 3, 10, and 17 from two particular IOLs, *Centro* and *Castelão*, to all other neighborhoods are shown in Fig. 4. In Fig. 4a,b, the distributions are represented in a polar coordinate system, where the radius stands for the shortest path, $D_{ij}^{(k)}$, and the angle stands for a random number between 0 and $2\pi$, for better visualization. We observe that $D_{ij}^{(k)}$ and $d_{ij}^{(k)}$ do not exhibit linear correlation, *i.e.*, a comparatively lower shortest path $D_{ij}^{(k)}$ between two neighborhoods does not necessarily imply that they are closer in the geographic space. As shown in Fig. 4c,d, we can also describe these distributions through the Kernel Density Estimation (KDE), defining an estimator $g(D_{ij}^{(k)})$ with a Gaussian kernel (see Methods). In both cases, we observe that $g(D_{ij}^{(k)})$ shifts to the right from week 3–10, indicating that there is, on average, an increase in $D_{ij}^{(k)}$, and shifts back to the left from week 10–17, partially recovering the urban mobility pattern observed in week 3. We find similar behaviors considering all other neighborhoods as IOLs, even in the cases where $g(D_{ij}^{(k)})$ has a bimodal-like shape, as shown in Fig. 4d. This shows that the restriction measures effectively resulted in the increase of the shortest path between the neighborhoods of Fortaleza.

### Spearman’s correlation of the closeness centrality

**Figure 5:**
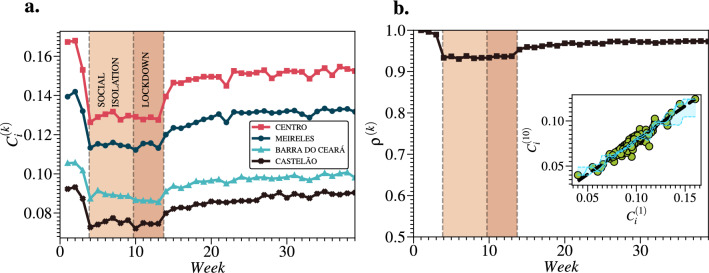
Closeness centrality and Spearman’s rank correlation coefficient. (**a**) We show the time evolution of the closeness centrality $C_i^{(k)}$ for some neighborhoods *i*. All $C_i^{(k)}$ curves exhibit a high similarity among themselves. Furthermore, they were synchronously affected by the restriction measures in the city of Fortaleza. (**b**) We also show the time evolution of Spearman’s rank correlation coefficient $\rho ^{(k)}$ between $C^{(k)}$ and $C^{(1)}$. We find that $\rho ^{(k)} > 0.92$ in all layers, which implies that the $C_i^{(k)}$ lines rarely cross each other. The inset shows a dashed black line that represents the linear relation between $C_i^{(1)}$ and $C_i^{(10)}$ (first week of lockdown) with a slope coefficient $a \approx 0.75$ ($r^2 \approx 0.90$). The solid blue line is the Nadaraya–Watson (NW) estimator, and the two dashed blue lines are its 95% Confidence Intervals (CIs) estimated using the bootstrap method. Such results prove that the restrictive measures were not only synchronous but also geographically homogeneous in Fortaleza.

As the network is a weakly connected graph for each temporal layer *k*, we calculate the time evolution of the closeness centrality, $C_i^{(k)}$, for all neighborhoods *i* (see Methods). In Fig. 5a., we observe that the values of $C_i^{(k)}$ were synchronously affected by the restriction measures, which resembles the $Z^{(k)}$ profile in Fig. 2a. For such metric, the most central neighborhood is *Centro* (downtown), while the least central is *Sabiaguaba*. Figure 5b shows the time evolution of the Spearman’s rank correlation coefficient^31^, $\rho ^{(k)}$, between the sets $C_i^{(k)}$ and $C_i^{(1)}$ (see Methods). We find that the $C_i^{(k)}$ lines rarely cross each other since $\rho ^{(k)} > 0.92$ in all layers. The inset shows the linear relation between $C_i^{(1)}$ and $C_i^{(10)}$ (first week of lockdown) with a slope coefficient $a \approx 0.75$ ($r^2 \approx 0.90$). Here, we use Nadaraya–Watson estimator to validate the linear relation between $C^{(k)}$ and $C^{(1)}$. We believe that these results are supporting evidence that the restriction measures were not only synchronous but also geographically homogeneous in Fortaleza. We conjecture that this can be explained in terms of the stability in the commuting patterns, *i.e.*, the restriction measures can increase the shortest path among the neighborhoods, but their proportional commuting patterns remain the same since there are strong socioeconomic ties between people and places within a city^32^. Furthermore, we also find that neighborhoods with high values of $C_i$ tend to exhibit unimodal-like shapes in $g(D_{ij}^{(k)})$ profiles, while the ones with low values tend to exhibit bimodal-like shapes.

### Arrival time and closeness centrality

**Figure 6:**
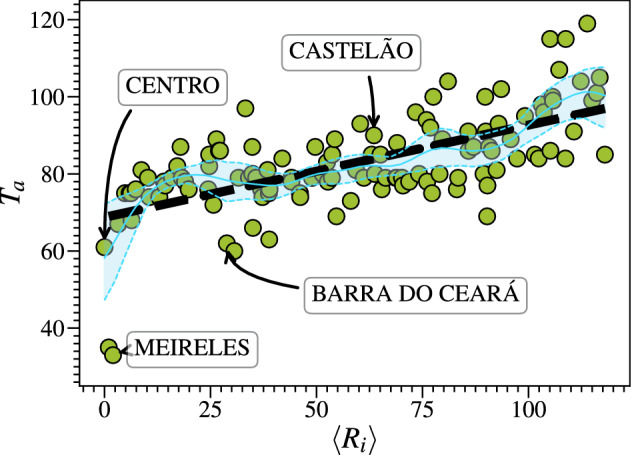
Disease arrival time $T_a$ and average rank position of the closeness centrality $\langle R_i \rangle$. We find a linear relation (dashed black line) between $T_a$ and $\langle R_{i} \rangle$ characterized by the slope coefficient $a \approx 0.24$ ($r^2 \approx 0.41$). Each point in the scatter plot is a neighborhood of the city of Fortaleza. The solid blue line is the Nadaraya–Watson (NW) estimator, and the two dashed blue lines are its 95% Confidence Intervals (CIs) estimated through the bootstrap method. We observe that the linear regression fitting follows the NW estimator and lies approximately within the CIs. This result reveals that the more neighborhoods are effectively central in the mobility network, the faster the disease arrives at them.

As shown in Fig. 6, the disease arrival time $T_{a}$ increases linearly with the average rank position of the closeness centrality, $\langle R_{i}\rangle$, calculated for all layers. This linear relation is characterized by the slope coefficient $a \approx 0.24$ ($r^2 \approx 0.41$). Again, we use Nadaraya–Watson estimator to validate the linear relation between $T_{a}$ and $\langle R_{i}\rangle$. Such result reveals that the more neighborhoods are effectively central in the mobility network, the faster the disease arrives at them. Consequently, as already shown in Fig. 2d, these neighborhoods also produce the highest number of reported cases throughout the investigated epidemic period.

## Conclusions

We have proposed a data-driven model to understand the relation between urban mobility and the spreading of COVID-19 in the city of Fortaleza, Ceará, Brazil. Our results show that the shortest bus rides within the city decreased $\approx 25$% more than the longest ones during the first epidemic wave of reported cases, *i.e.*, from March to December 2020. Such a finding is the opposite of what has been observed at the intercity scale^10^. Applying the proposed model, we found that mobility changes among the neighborhoods are synchronous since their closeness centrality curves have a similar shape during the studied period. They can also be considered geographically homogeneous because there are almost no crossings among these curves. Both behaviors are also uncommon to see on a larger geographic scale - the former due to different stages of local outbreaks and the latter due to the invariance of the commuting patterns within a city, which can be explained by the fact that there are strong socioeconomic ties between people and places. Finally, we found that the most central neighborhoods in the mobility network were not only the primary focuses of the COVID-19 outbreak but also the top neighborhoods in the number of reported cases at the end of epidemic wave. We emphasize that our mobility dataset is based on public bus transport only. However, as explained in SI, it is a good proxy for the total urban mobility of Fortaleza. Another limitation that can be pointed out in our study is that there was indeed a sub-notification of reported cases of COVID-19 in Fortaleza. However, our results suggest the existing bias is not strong enough to change our conclusions. As a perspective, the development of dynamic models could improve the understanding of the studied phenomenon at the intra-city scale. Despite the leading role of massive vaccination campaigns in controlling outbreaks of respiratory communicable diseases and the large number of studies published in the last three years regarding the use of Non-Pharmaceutical Interventions (NPIs) to mitigate the spread of COVID-19, more research is still needed to improve public policies based on NPIs, mainly for situations that, for some reason, there are no vaccines available, which usually happen in the least developed and developing countries. Historically, most local outbreaks in Fortaleza were controlled through massive vaccination campaigns, *e.g.*, in the cases of H1N1 and measles^33,34^. In such cases, where both were vaccine-preventable diseases, the main interventions were the characterization of transmission chains to interrupt the spread (especially in the case of measles), and large-scale vaccination, which included an active search for unvaccinated individuals. Furthermore, focused quarantines were only recommended when outbreaks were in institutions such as schools, prisons, and churches. We believe that these results may unveil new strategies to impose restriction measures in the cities guiding decision-makers with smart actions in public health policies, as well as supporting future research on urban mobility and epidemiology.

## Methods

### Datasets

#### Population

We use the 2010 census population provided by the Brazilian Institute of Geography and Statistics (IBGE)^35^ to define the number of inhabitants from the 119 neighborhoods of the city of Fortaleza, Ceará, Brazil.

#### Reported cases and deaths of COVID-19

We collect the number of reported cases and deaths of COVID-19 from Fortaleza in IntegraSUS^26^, the official repository of the Ceará State Government, and make them available (see Data Availability). For both, the file structure is similar: each row represents the number of reported cases or deaths of COVID-19 on a specific date for all neighborhoods. We emphasize that such a dataset is retrospectively updated and corrected.

#### Human development index

The Fortaleza City Hall made available the Human Development Index (HDI) by neighborhood. (see Data Availability)

#### Urban mobility

The Fortaleza City Hall made available a set of 39 weekly Origin-Destination (OD) matrices $\{M\}$, based on bus validations during weekdays, among all neighborhoods of Fortaleza (see Data Availability). For each week *k*, $M_{ij}^{(k)}$ represents the average of the number of individuals that commute from the neighborhood *i* to the neighborhood *j*, *i.e.*, $M_{ij}^{(k)}$ only considers one-way rides. The period of the data ranges from March to December 2020. We emphasize that matrices *M* are identical to matrices *F*, except for the main diagonals, since $M_{ii}^{(k)}$ stands for the number of intra-neighborhood rides, while $F_{ii}^{(k)} = N_i - \sum _{j(i\ne j)} F_{ij}^{(k)}$ stands for the number of inhabitants that remain in the neighborhood *i*. Finally, we also note that the neighborhood *Vila Ellery* has no entries in *M* during the studied period. For this reason, we remove it from the entire analysis.

### The model

**Figure 7:**
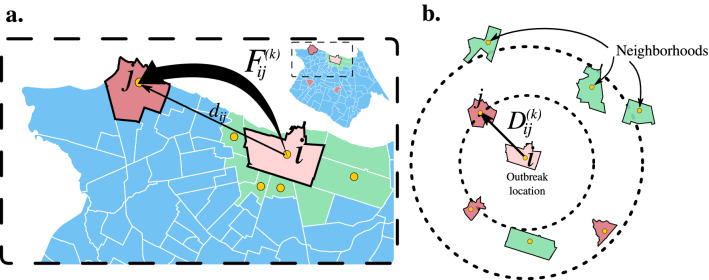
Geographic distance and shortest path distance based on urban mobility. (a) The geographic distance $d_{ij}$ from a neighborhood *i* (light red), a hypothetical Initial Outbreak Location (IOL), to a neighborhood *j* (red for far and light green for close neighborhoods) is illustrated in the Fortaleza map. It is also shown $F_{ij}^{(k)}$, the average daily number of individuals that ride from *i* to *j* at week *k*. (b) The neighborhood *i* is set in the origin of a polar coordinate system, where the radius stands for the magnitude of the shortest path $D_{ij}^{(k)}$ from *i* to *j* at week *k* and the angle stands for a random number between 0 and $2\pi$, for better visualization. We emphasize that the neighborhood *j*, which is geographically far away from neighborhood *i* may, however, be *effectively* close to it (low $D_{ij}^{(k)}$), *i.e.*, may have high commuting levels with *i*. Furthermore, the opposite situation can also happen. All regional maps were produced using Python packages. The map is from the package Matplotlib Version 3.7 (https://matplotlib.org/).

We investigate the effects of the restriction measures on the urban mobility of Fortaleza by representing the data in terms of a multiplex network^36^ with 39 layers, each corresponding to a week during the period from March to December 2020. Precisely, for each week *k*, the vertices *i* stand for the $n=118$ neighborhoods of Fortaleza, while the weighted directed edges $(i,j,w_{ij}^{(k)})$ stand for the commuting patterns from neighborhoods *i* to others *j*. The weight $w_{ij}^{(k)}$ is given by^16^:

1 $$\begin{aligned} w_{ij}^{(k)} = 1-\ln \frac{F_{ij}^{(k)}}{N_i}, \end{aligned}$$

where $F_{ij}^{(k)}$ is the average daily number of individuals that ride from *i* to *j* at week *k*, and $N_i$ is the population of *i*. We emphasize that $F_{ij}^{(k)}$ is estimated from the public urban transportation using a dataset with more than 107 million bus validations (see Datasets). Furthermore, the main diagonal elements of the matrices $F^{(k)}$ are set as $F_{ii}^{(k)} = N_i - \sum _{j(i\ne j)} F_{ij}^{(k)}$. Figure 7a,b show a schematic representation of the geographic distance $d_{ij}$ between the centroids of the neighborhoods *i* and *j*, as well as the shortest path (or the effective distance) $D_{ij}^{(k)}$ from *i* to *j* at the week *k*, respectively. The distance $d_{ij}$ is calculated through the Haversine formula^37^, while $D_{ij}^{(k)}$ is calculated through the Dijkstra’s algorithm^38^. As highlighted in light red in Fig. 7, a neighborhood *j* which is geographically far away from neighborhood *i* may, however, be *effectively* close to it (low $D_{ij}^{(k)}$), *i.e.*, may have high commuting levels with *i*.

### Closeness centrality

As all layers of the proposed multiplex network are directed subgraphs, the closeness centrality $C_i^{(k)}$ of the neighborhood *i* in the layer *k* is given by^36^,

2 $$\begin{aligned} C_i^{(k)} = \frac{n-1}{\sum _j D_{ji}^{(k)}}, \end{aligned}$$

where $n-1$ is the number of neighborhoods *j* that reach *i*, and $D_{ji}^{(k)}$ is the shortest path from *j* to *i*, *i.e.*, $D_{ji}^{(k)}$ is the inward distance of *i*.

### Spearman’s rank correlation

The Spearman’s rank correlation coefficient^31^, $\rho _i^{(k)}$, between $C_i^{(k)}$ and $C_i^{(0)}$ is calculated as follows:

3 $$\begin{aligned} \rho _i^{(k)} = \frac{\text {cov}(R_i^{(k)},R_i^{(0)})}{\sigma (R_i^{(k)})\sigma (R_i^{(0)})}, \end{aligned}$$

where $R_i^{(k)}$ is the ranking of $C_i^{(k)}$, $\text {cov}$ is the covariance and $\sigma$ is the standard deviation.

### Nadaraya–Watson estimator

The Nadaraya–Watson (NW) estimator is defined by the following kernel smoother function^29,30^:

4 $$\begin{aligned} \hat{m}_h (x) = \frac{\sum _{i=1}^N K_h(x-X_i)Y_i}{\sum _{i=1}^N K_h(x-X_i)}, \end{aligned}$$

where *x* is the evaluation point, *N* is the number of points of the data distribution $\{X_i,Y_i\}$, $K_h(x-X_i) = \exp [(x-X_i)^2/(2h^2)]$ is a Gaussian kernel, and *h* is the bandwidth. We estimate the bandwidth *h* by the least squares cross-validation method^39,40^. We compute the 95% ($\alpha = 0.05$) Confidence Intervals (CIs) over 500 random bootstrapping samples with replacement through the so-called $\alpha /2$ quantile function.

### Kernel density estimator

The Kernel Density Estimation (KDE) is a non-parametric method to estimate the probability density function based on kernel smoothing^41,42^. Given a data distribution $\{X_i\}$ with *N* points, the estimator is calculated as follows:

5 $$\begin{aligned} g(x) = \frac{1}{N}\sum _{i=1}^N K_h(x-X_i), \end{aligned}$$

where *x* and $K_h(x-X_i)$ are defined in a way similar to the NW estimator. On the other hand, the bandwidth *h* is defined by Scott’s rule ($h=N ^{-1/5}$).

## Supplementary Information

Supplementary Information.

## Data Availability

The data that support the findings of this study are available in Zenodo with the identifier https://doi.org/10.5281/zenodo.7655082.
