# Supplementary material for: Unveiling the paths of COVID-19 in a large city based on public transportation data
Source: Sci Rep. 2023 Apr 8;13:5761. doi: 10.1038/s41598-023-32786-z (PMC10082688; doi:10.1038/s41598-023-32786-z)
Supplement: Supplementary file 1 — Supplementary Information. [file 41598_2023_32786_MOESM1_ESM.pdf]

Supplementary Information: Unveiling the paths of COVID-19 in a large city based on public transportation data

Jorge L. B. Araújo[1*], Erneson A. Oliveira[1,2,3], José S. Andrade Jr.[4], Antonio S. Lima Neto[5,6], Vasco Furtado[2,7]

[1] *Laboratório de Ciência de Dados e Inteligência Artificial, Universidade de Fortaleza, Fortaleza, Ceará, 60811-905, Brasil.*

[2] *Programa de Pós Graduação em Informática Aplicada, Universidade de Fortaleza, Fortaleza, Ceará, 60811-905, Brasil.*

[3] *Mestrado Profissional em Ciências da Cidade, Universidade de Fortaleza, Fortaleza, Ceará, 60811-905, Brasil.*

[4] *Departamento de Física, Universidade Federal do Ceará, Fortaleza, Ceará, 60455-760, Brasil*

[5] *Célula de Vigilância Epidemiológica, Secretaria Municipal da Saúde, Fortaleza, Ceará, 60810-670, Brasil.*

[6] *Centro de Ciências da Saúde, Universidade de Fortaleza, Fortaleza, Ceará, 60811-905, Brasil.*

[7] *Empresa de Tecnologia da Informação do Ceará, Governo do Estado do Ceará, Fortaleza, Ceará, 60130-240, Brasil.*

[*] *Correspondence to: jorgearaujo@unifor.br*

(Dated: February 21, 2023)

I. ACCUMULATED CASES AND DEATHS OF COVID-19

In 2020, cases and deaths of COVID-19 were registered in all neighborhoods of Fortaleza (see Fig. 1). Figure SI-1 shows the expected linear relation between accumulated cases and deaths. This linear behavior, also identified by the CI of the Nadaraya-Watson estimator, reveals a slope $a \approx 11.3$ ($r^2 \approx 0.24$). Thus, an effective ratio of ≈ 88 deaths for 1000 cases was observed in the neighborhoods of Fortaleza in 2020.

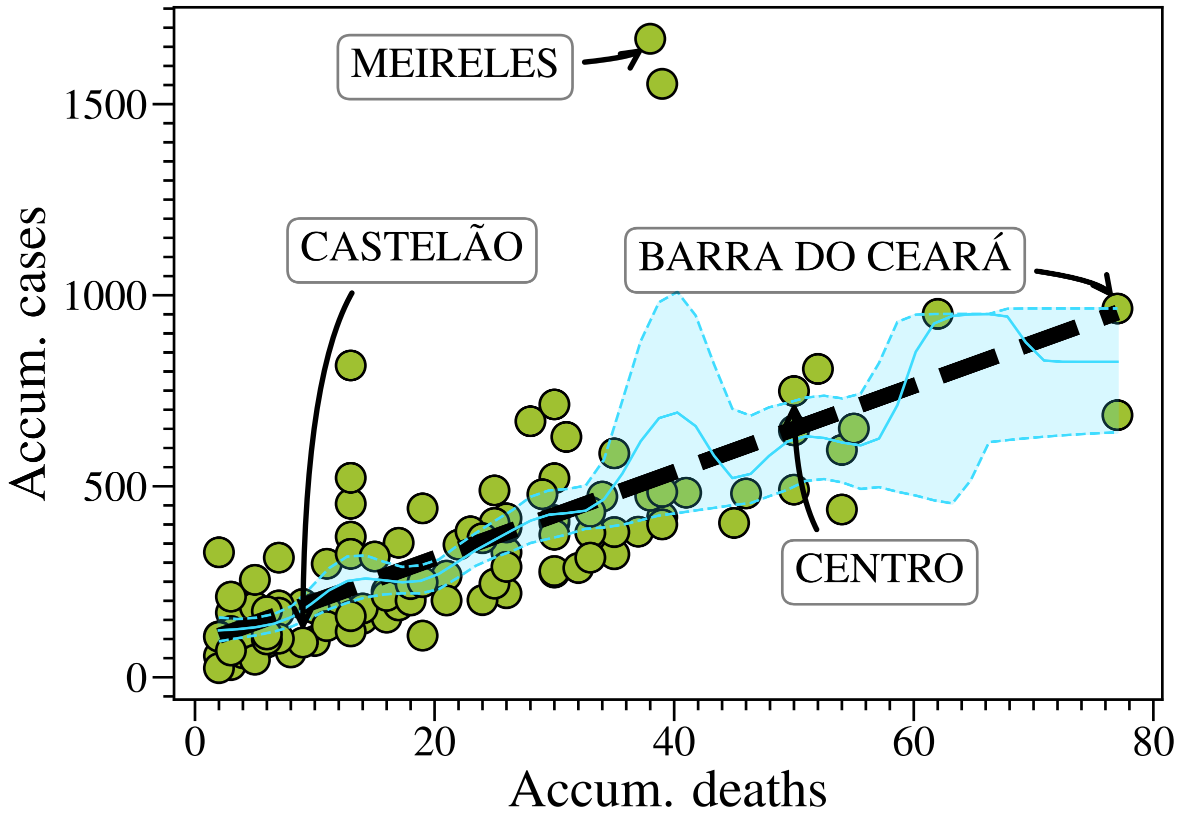

Fig. SI-1. Accumulated cases versus accumulated deaths of COVID-19. Each point represents a neighborhood of the city of Fortaleza. The dashed black line is a linear regression with slope $a \approx 11.3$ ($r^2 \approx 0.24$). The solid blue line is the Nadaraya-Watson (NW) estimator, and the two dashed blue lines are its 95% Confidence Intervals (CIs) estimated through the bootstrap method. This result reveals a ratio of approximately ≈ 88 deaths for 1000 cases in the neighborhoods of Fortaleza.

II. MOBILITY CHANGES AND HUMAN DEVELOPMENT INDEX

We show the mobility changes in lockdown (week 10 in relation to week 1) against the Human Development Index (HDI) of the neighborhoods of Fortaleza in Fig. SI-2. Here, $Z_i^{(k)} = \sum_{j(j\neq i)} F_{ij}$ is the number of trips to and from neighborhood i in a week k. We find that neighborhoods with higher HDI have greater decreases in urban mobility. This relation was also observed in previous studies [1, 2], reinforcing the importance of socioeconomic measures in the mobility change profile and, consequently, in how COVID-19 spreads.

III. GOOGLE MOBILITY DATASET

Urban mobility calculated only through the flow of individuals who use public transport can create a bias in the proposed analysis due to the limitation of using only one mobility modal. In Fig. SI-3, we show the mobility change Z^*, based on cellphone data provided by Google [3], for *Transit stations*, *Workplaces*, *Retail and recreation*, *Grocery and pharmacy*, *Parks*, and *Residential*. We have rescaled the time axis for better comparison with the time series presented in the main text, where week $k = 1$ refers to March 1st. We can check the representativeness of the mobility dataset by making a comparison between the mobility change Z, based on public bus transportation, and the mobility change Z^* for the same period in Fortaleza (see Fig. 2 and Fig. SI-3, respectively). We find both exhibit similar mobility patterns, which suggests that the mobility dataset used in this study is a good proxy for the total urban mobility of Fortaleza.

IV. ANALYSIS OF THE BEST THRESHOLD n_{ac}^*

Figure SI-4 shows the analysis of the best threshold n_{ac}^* for the number of accumulated reported cases n_{ac}. Given a neighborhood i, labeled as the Initial Outbreak Location (IOL), we calculate the Pearson correlation coefficient [4]

$$r_i = \frac{\mathrm{cov}(T_a, D)}{\sigma(T_a)\sigma(D)}, \tag{1}$$

where T_a is the arrival time of the diseases in neighborhood j, D is the shortest path distance from all other neighborhoods j to i, cov is the covariance and σ is the standard deviation. The D is calculated taking into account the average of the weekly Origin-Destination (OD)

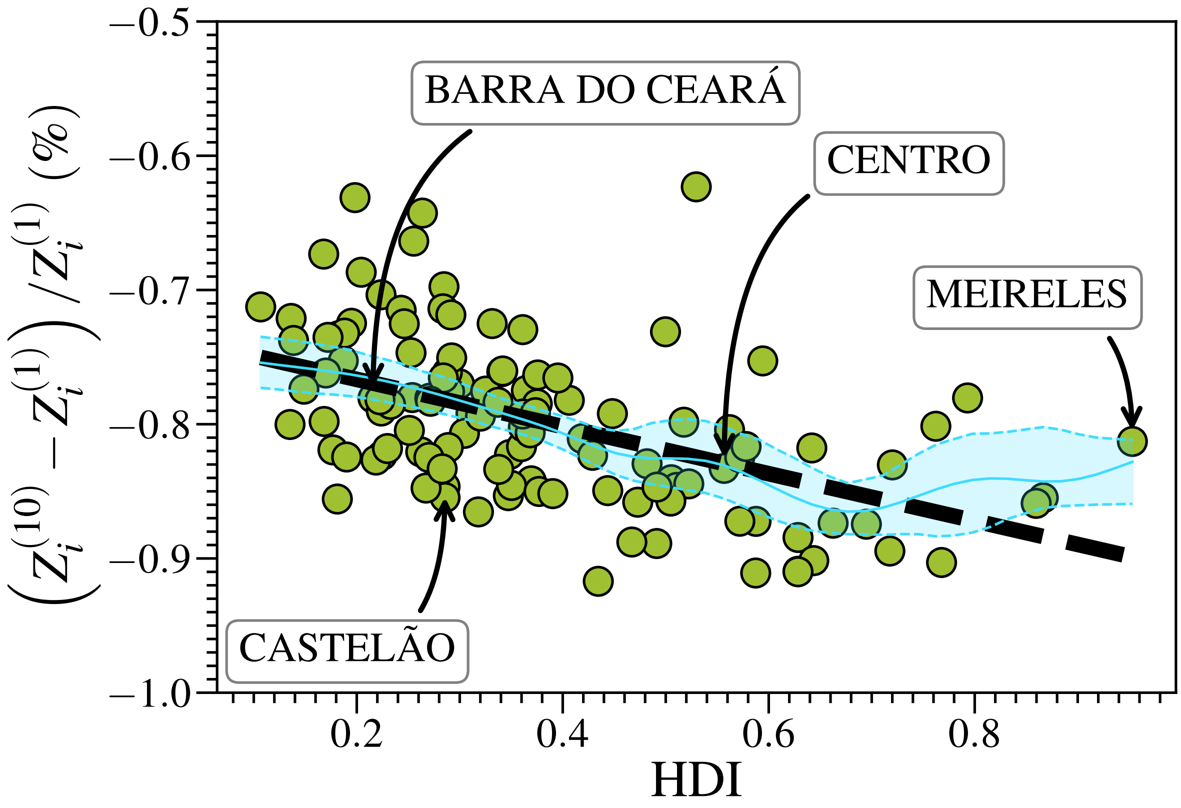

Fig. SI-2. Mobility changes as a function of the Human Development Index (HDI). Here, $Z_i^{(k)} = \sum_{j(j\neq i)} F_{ij}$ is the number of trips to and from neighborhood i in a week k. We compare the mobility profile in the week $k = 10$ (lockdown) with the baseline $k = 1$ (*pre-pandemic*). Each point on the scatter plot represents a neighborhood of Fortaleza. The dashed black line is a linear regression with slope $a \approx -0.17$ ($r^2 \approx 0.30$). The solid blue line is the Nadaraya-Watson (NW) estimator, and the two dashed blue lines are its 95% Confidence Intervals (CIs) estimated through the bootstrap method. We found that neighborhoods with higher HDI had greater decreases in urban mobility during the first wave caused by COVID-19.

matrices $M^{(k)}$ from $k = 1$ to $k = 10$, *i.e.*, until the week that the disease reached the last neighborhood for $n^*_{ac} = 20$. Figure SI-4(a) shows the boxplot representation of the r distribution for all possible IOL versus the several values of n^*_{ac}. Therefore, we choose $n^*_{ac} = 6$ as the best threshold value because it is the value that establishes a plateau and maximizes the median of the r distribution. We also show the spatial distribution of the

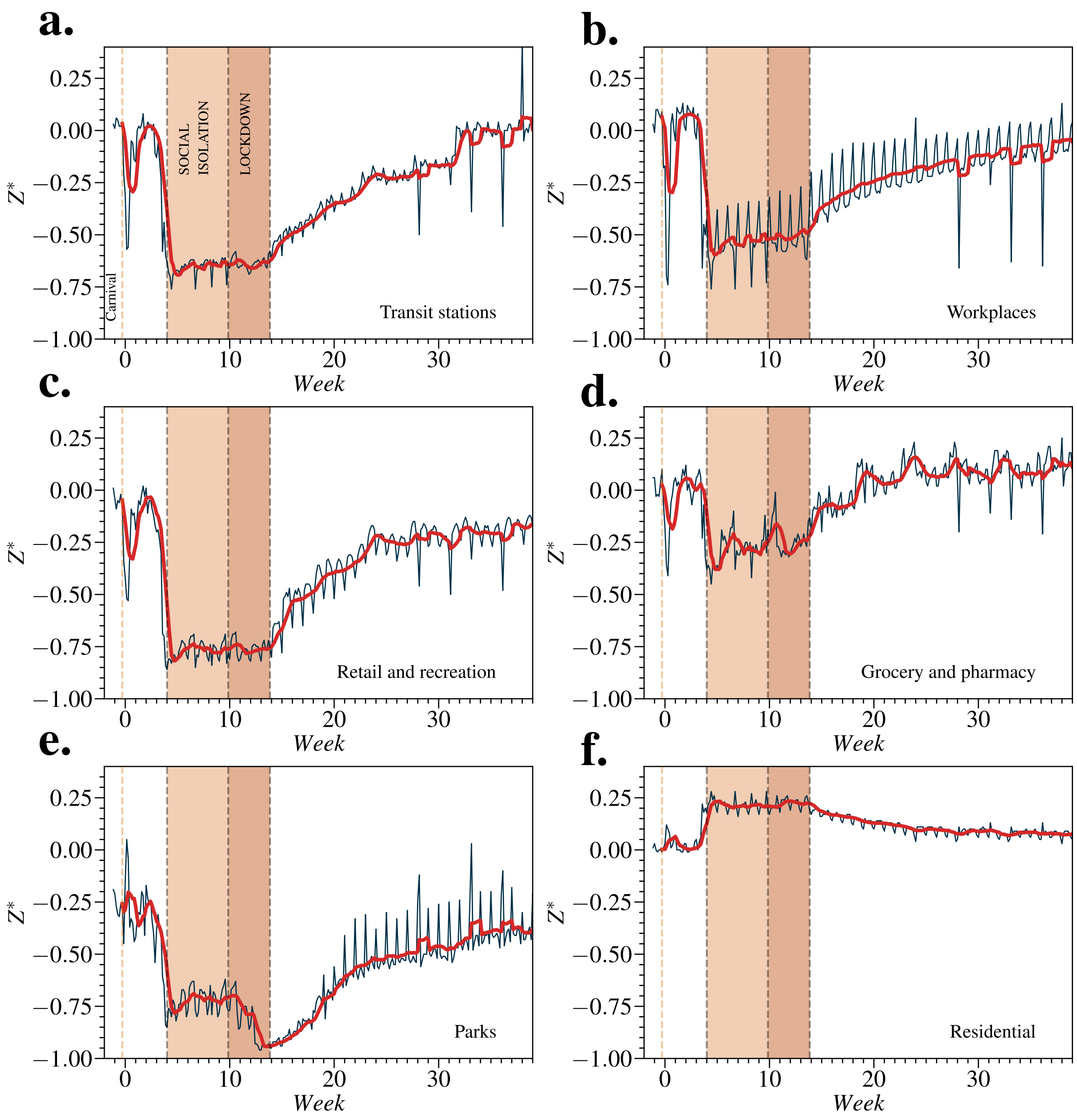

Fig. SI-3. Mobility change Z^* for the city of Fortaleza through google mobility dataset. We rescaled the time axis for better comparison with the time series presented in the main text, where week $k = 1$ refers to March 1st. Mobility change profiles are presented for (a) *Transit stations*, (b) *Workplaces*, (c) *Retail and recreation*, (d) *Grocery and pharmacy*, (e) *Parks*, and (f) *Residential*. Brown zones refer to periods of social isolation and lockdown. The dashed orange line highlights the Carnival (February 10th). The red curves are seven-day moving averages.

Pearson correlation coefficient r for $n^*_{ac} = 6$ in Fig. SI-4(b). We also note that the most likely neighborhoods to have started the first COVID-19 outbreak in the city of Fortaleza are those from the northern region of the city. Such a result is consistent with observational data.

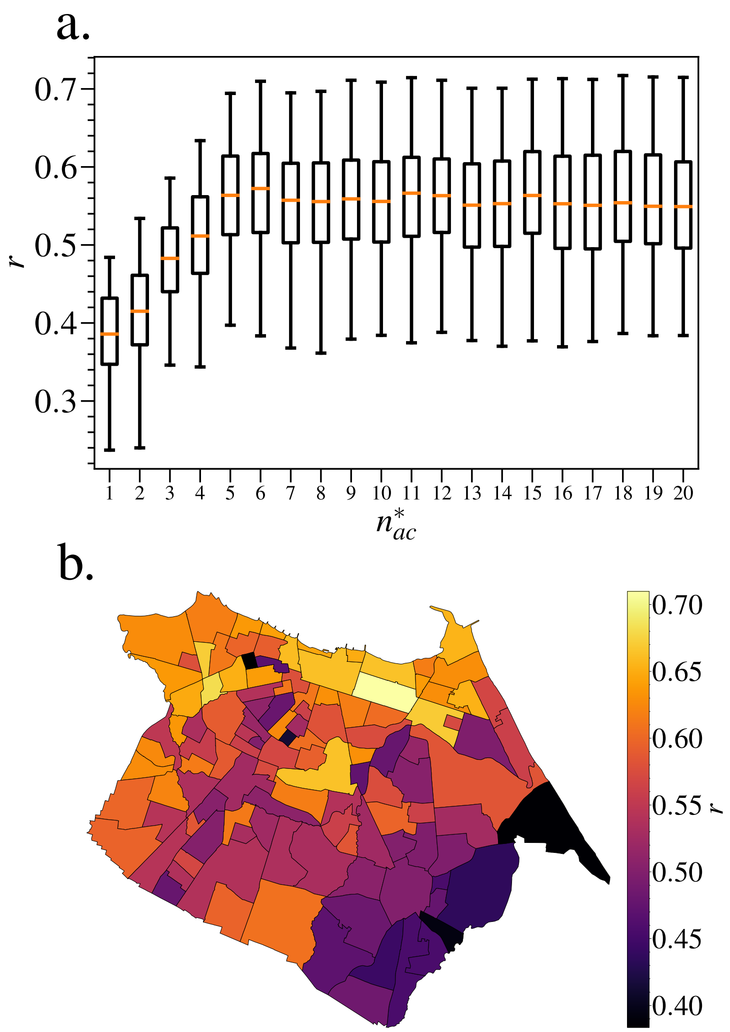

Fig. SI-4. **Analysis of the best threshold** n^*_{ac}. In (a), the boxplot shows the distribution of the Pearson correlation coefficient r versus the number of accumulated reported cases n_{ac}. The median and the 25th and 75th percentiles of each distribution are represented by the orange lines and by the limits of the boxes, respectively. We choose $n^*_{ac} = 6$ as the best threshold value because it is the value that establishes a plateau and maximizes the median of the r distribution. In (b), the map shows the spatial distribution of r for $n^*_{ac} = 6$.

[1] Gozzi, N., Tizzoni, M., Chinazzi, M. et al. Estimating the effect of social inequalities on the mitigation of COVID-19 across communities in Santiago de Chile. Nat Commun 12, 2429 (2021). doi: 10.1038/s41467-021-22601-6.

[2] Laetitia Gauvin, Paolo Bajardi, Emanuele Pepe, Brennan Lake, Filippo Privitera and Michele Tizzoni. Socio-economic determinants of mobility responses during the first wave of COVID-19 in Italy: from provinces to neighbourhoods. Royal Society (2021). doi: 10.1098/rsif.2021.0092.

[3] Google LLC. Google COVID-19 Community MObility Reports. 2021. Available from `https://www.google.com/covid19/mobility/`

[4] Kutner MH, Nachtsheim CJ, Neter J, Li W. Inferences in regression and correlation analysis. In: Applied Linear Statistical Models. 5th ed. Singapore: McGraw-Hill/Irvin, 2005.
